# *In-silico* comparison of two induction regimens (7 + 3 vs 7 + 3 plus additional bone marrow evaluation) in acute myeloid leukemia treatment

**DOI:** 10.1186/s12918-019-0684-0

**Published:** 2019-01-31

**Authors:** Jan Christoph Banck, Dennis Görlich

**Affiliations:** 1 Institute of Biostatistics and Clinical Research, Westfälische Wilhelms-Universität Münster, Münster, Germany; 2 Department of Medicine III, University Hospital, Ludwig-Maximilians-Universität Munich, Munich, Germany

**Keywords:** Acute leukemia, Mathematical model, Systems medicine, First line induction therapy

## Abstract

**Background:**

Clinical integration of systems biology approaches is gaining in importance in the course of digital revolution in modern medicine. We present our results of the analysis of an extended mathematical model describing abnormal human hematopoiesis. The model is able to describe the course of an acute myeloid leukemia including its treatment. In first-line treatment of acute myeloid leukemia, the induction chemotherapy aims for a rapid leukemic cell reduction. We consider combinations of cytarabine and anthracycline-like chemotherapy. Both substances are widely used as standard treatment to achieve first remission. In particular, we compare two scenarios: a single-induction course with 7 days cytarabine and 3 day of anthracycline-like treatment (7 + 3) with a 7 + 3 course and a bone marrow evaluation that leads, in case of insufficient leukemic cell reduction, to the provision of a second chemotherapy course. Three scenarios, based on the leukemias growth kinetics (slow, intermediate, fast), were analyzed. We simulated different intensity combinations for both therapy schemata (7 + 3 and 7 + 3 + evaluation).

**Results:**

Our model shows that within the 7 + 3 regimen a wider range of intensity combinations result in a complete remission (CR), compared to 7 + 3 + evaluation (fast: 64.3% vs 46.4%; intermediate: 63.7% vs 46.7%; slow: 0% vs 0%). Additionally, the number of simulations resulting in a prolonged CR was higher within the standard regimen (fast: 59.8% vs 40.1%; intermediate: 48.6% vs 31.0%; slow: 0% vs 0%). On the contrary, the 7 + 3 + evaluation regimen allows CR and prolonged CR by lower chemotherapy intensities compared to 7 + 3. Leukemic pace has a strong impact on treatment response and especially on specific effective doses. As a result, faster leukemias are characterized by superior treatment outcomes and can be treated effectively with lower treatment intensities.

**Conclusions:**

We could show that 7 + 3 treatment has considerable more chemotherapy combinations leading to a first CR. However, the 7 + 3 + evaluation regimen leads to CR for lower therapy intensity and presumably less side effects. An additional evaluation can be considered beneficial to control therapy success, especially in low dose settings. The treatment success is dependent on leukemia growth dynamics. The determination of leukemic pace should be a relevant part of a personalized medicine.

**Electronic supplementary material:**

The online version of this article (10.1186/s12918-019-0684-0) contains supplementary material, which is available to authorized users.

## Background

Acute myeloid leukemia (AML) is a rare malignant disease of the blood cell formation and is the most common acute leukemia among adults leading to most death events caused by leukemias [1]. In particular, AML is constituted of genetically different hematopoietic neoplasms that collectively stem from various multistep mutations affecting the myeloid cell line resulting in accumulating neoplastic precursor cells [2]. The intrinsic origin of AML is a small subset of leukemic stem cells (LSC) leading to a self-perpetuating proliferation of clonal progenitor cells also referred to as blasts [3]. Fast growing number of inoperative and undifferentiated blasts induce a disorder of normal hematopoiesis located in the bone marrow with further systemic implications in blood and other tissues [1]. As part of a clonal evolution in one patient, genetically different AML clones exist, develop and are specifically responsible for diagnosis or potential relapse because of a presumed selection by chemotherapy [4].

This complex pathogenesis and additional resistance mechanisms lead to various treatment strategies and respective various patient outcomes [5, 6]. Independently from new more tailored treatment approaches (e.g. CAR-T cells), an established but relatively unspecific combination chemotherapy of cytarabine and anthracycline sets still the standard aiming for a first clinical remission during induction therapy [2, 5].

A widely used therapeutic approach is the 7 + 3 regimen (starting with seven days cytarabine and supplementary for the first three days anthracycline). In clinical practice variation of this regimen exists differing e.g. in dose and/or schedule [5, 7]. Different 7 + 3 regimens are preferred depending on respective region, e.g. an evaluation process with potential re-induction in the United States compared to a preferred double induction in Europe [8].

Our scientific objectives were to compare different intensities of two 7 + 3 chemotherapy regimens using a mathematical model, which characterizes the dynamics of AML using ordinary differential equations. We primarily intend to increase the efficiency of this known induction therapy by detecting more disease-specific treatment conditions. A single-induction 7 + 3 regimen was compared with a 7 + 3 regimen plus an additional bone marrow (BM) evaluation on day 14 and/or 21 after treatment start with a potential second induction cycle.

In total, we analyzed about ten thousand different intensity combinations, which is more than in tangible experiments (in vivo or in vitro) or clinical studies are feasible [9–11].

To evaluate each scenario, we computed the time from treatment start to complete remission (CR) and the following CR duration as two essential clinical parameters that enable a rational comparison [12].

## Methods

For our analysis, we extended the published two-compartment AML model by Stiehl et al. [13]. The two compartments represent hematopoietic stem cells (HSC) within the bone marrow (first compartment) which can differentiate by cell division into non-proliferating (differentiated) cells (second compartment). Healthy and non-healthy (leukemic stem cells, LSC) cells are modelled separately and differ in their parameter values. The model is able to explain the dynamics of cell population abundance adequately [13–16]. Normal HSC and pathological LSC are represented by a set of two ordinary differential equations. Cellular abundance (in cells/kg body weight) at day *t* is denoted by *c*_1_(*t*) for HSC, *c*_2_(*t*) for healthy differentiated cells, *l*_1_(*t*) for LSC and *l*_2_(*t*) for non-proliferating leukemic cells, respectively. HSC, LSC and non-proliferating leukemic cells are considered to reside in the bone marrow, whereas healthy differentiated cells belong to the blood stream. This model assumption corresponds with prior results by Stiehl [13], who showed that a model extension by a bone marrow exit did not lead to qualitative changes of the selected cell properties via chemotherapy. The proliferation rates *p*^*c*^ (HSC) and *p*^*l*^ (LSC) and the self-renewal rates, (*a*^*c*^ for HSC, *a*^*l*^ for LSC), are used to characterize healthy hematopoiesis and leukemia growth kinetics, respectively. In the model the term of self-renewal represent this self-sustaining fraction as a proportion (0–1). We considered proliferation rates in the range 0–2 and self-renewal rates between 0 and 1.

Depletion (e.g. apoptosis or migration into, for our model, negligible states) of non-proliferating cells is modelled as constant death rate ${d}_2^c$ and${d}_2^l$. Resident cells (here *c*_1_, *l*_1_, and *l*_2_) will also deplete, if bone marrow cell number exceed a density threshold value, i.e. the physiological equilibrium value of bone marrow cell count. The function *d*(*x*(*t*)) is an additional death rate describing the fraction of bone marrow cells dying because of overcrowding. A feedback regulation denoted by *s*(*t*) is integrated to represent cellular communication. Affecting self-renewal, feedback regulation leads to the result that increasing number of differentiated healthy cells causes a reduced number of HSC and LSC (and vice versa).

Intensive induction chemotherapy of AML contains a combination of two or more chemotherapeutics applied in a specific therapy regimen [5, 7]. We extended the model to implement the 7 + 3 scheme based on 7 days cytarabine and 3 days anthracycline treatment. Cytarabine acts as an antimetabolic agent and attacks primarily on cells during their synthesis phase (S-phase) by inhibiting the DNA-polymerase α [17, 18]. The modelled chemotherapy mechanism (*k*_*cyt*_ · *p*^*c*^ · *c*_1_(*t*)) can be considered as a cytarabine-like chemotherapy acting on proliferating cells. Anthracycline affects proliferating and non-proliferating cells via various mechanisms (e.g. inhibition of topoisomerase II or free radical generation) [19]. A second chemotherapy mechanism has been introduced acting on non-proliferating cells. We assume that the effect of anthracycline on mitotic cells is limited to the proliferation phase. This is justified by experimental observation that anthracycline’s toxicity on mitotic cells is mainly due to active proliferative state [20–22]. The extended Stiehl model [13] is defined by

1 $$ \frac{d}{dt}{c}_1(t)=2\cdot {a}^c\cdot {p}^c\cdot s(t)\cdot {c}_1(t)-{p}^c\cdot {c}_1(t)-d\left(x(t)\right)\cdot {c}_1(t)-{k}_{cyt}\cdot {p}^c\cdot {c}_1(t)-{k}_{anthra}\cdot {p}^c\cdot {c}_1(t) $$

2 $$ \frac{d}{dt}{c}_2(t)=2\cdot {p}^c\cdot {c}_1(t)-2\cdot {a}^c\cdot {p}^c\cdot s(t)\cdot {c}_1(t)-{d}_2^c\cdot {c}_2(t)-{k}_{anthra}\cdot {c}_2(t) $$

3 $$ \frac{d}{dt}{l}_1(t)=2\cdot {a}^l\cdot s(t)\cdot {p}^l\cdot {l}_1(t)-{p}^l\cdot {l}_1(t)-d\left(x(t)\right)\cdot {l}_1(t)-{k}_{cyt}\cdot {p}^l\cdot {l}_1(t)-{k}_{anthra}\cdot {p}^l\cdot {l}_1(t) $$

4 $$ \frac{d}{dt}{l}_2(t)=2\cdot {p}^l\cdot {l}_1(t)-2\cdot {a}^l\cdot s(t)\cdot {p}^l\cdot {l}_1(t)-{d}_2^l\cdot {l}_2(t)-d\left(x(t)\right)\cdot {l}_2(t)-{k}_{anthra}\cdot {l}_2(t) $$

5 $$ x(t)={c}_1(t)+{l}_1(t)+{l}_2(t) $$

6 $$ s(t)=\frac{1}{1+{k}^c{c}_2(t)} $$

7 $$ d\left(x(t)\right)={10}^{-10}\cdotp \max \left(0,x(t)-4\cdotp {10}^9 cells/ kg\right) $$

The model represents an intermediate state between Model 1 and Model 2 from [13]. It is known that the bulk of leukemic cells express granulocyte colony-stimulating factor (G-CSF) receptors [23]. G-CSF is the main mediator for hematopoietic feedback regulation and can stimulate as well leukemic cells [24]. G-CSF-feedback regulation is mainly directed by, not fully understood, transcriptional signaling processes with STAT3/SOCS proteins [25–27]. A relevant amount of AML subtypes shows a significant dysregulation of STAT3/SOCS-related pathways [28–31]. Thus, we assume for a wider part of AML no negative G-CSF feedback regulation by leukemic cells. As an implementation the leukemic cell self-renewal depends on the feedback *s(t)* (eq. (6)), but the feedback *s(t)* does not depend on the leukemic cell count in our model.

We implemented the model in the statistical software R [32]. Numerical solutions for the ordinary differential equations were calculated using the R package ‘deSolve’ [33]. A more detailed description, including the parametrization of the model, is given in Additional file 1. The R syntax is given in Additional file 2.

To analyze the model, we performed the following steps

### Identification of leukemic pace

Different leukemias (characterized by self-renewal, *a*^*l*^, and proliferation rate, *p*^*l*^) from initial prediagnostic occurrence (i.e. a model state which, in real patients, is below the diagnostic threshold) to the onset of leukemia were simulated. When the leukemia hits the diagnosis threshold (20% blasts, following international guidelines [34]) treatment is triggered. Blast fraction is defined as $\frac{l_1+{l}_2}{c_1+{l}_1+{l}_2\ }$ . We assume that in bone marrow there is no routine distinction made between LSC and non-proliferating leukemic cells [35]. Indeed, in clinical practice this distinction could be drawn for further investigation [36, 37].

### Selection of leukemia

We consider one distinct leukemic (parameter combination) per patient and identified three parameter sets, one leading to slow, one leading to intermediate and one leading to fast pace (cp Fig. 3 and Table 2). These parameter combinations can e.g. result in a leukemia emerging from different genetic characteristics. We use these parameter sets for our simulations to study how the growth kinetics (leukemic pace) influences outcome after induction therapy.

### Simulation of two combination therapy regimens

**Fig. 1:**
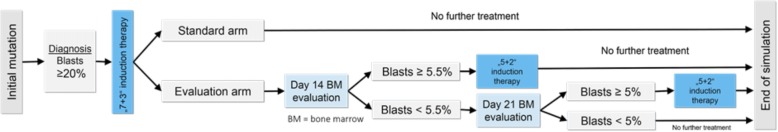
Study design. We simulated two different therapy regimens (study arms). The standard arm contains a single induction therapy using the 7 + 3 regimen (7 days cytarabine + 3 days anthracycline). After the induction the course of the leukemia is observed without further intervention. The evaluation arm contains one or two additional bone marrow (BM) evaluations. Based on the blasts abundance (%) a second induction consisting of a 5 + 2 protocol can be given. Patients with blast clearance will be observed without further intervention

Two chemotherapy regimens of AML have been modelled and analyzed (Fig. 1).

All simulations start with a small amount (1 cell / kg) in the L1 compartment and trajectories are observed. The initial state of the simulations is given in Table 1. When the blasts percentage hits the diagnostic threshold, therapy is triggered.

**Table 1:** Initial condition for all simulations

Compartment	Initial state
*c*_1_(*t* = 0)^a^	2 × 10^9^ cells/kg
*c*_2_(*t* = 0)^a^	3.9 × 10^9^ cells/kg
*l*_1_(*t* = 0)	1 cell/kg
*l*_2_(*t* = 0)	0 cells/kg

^a^Compartment *c*_*1*_ and *c*_*2*_ start in the steady state of the healthy systems (without leukemic cells or therapy)

The “standard arm” resembles a single 7 + 3 induction course [38] with a cytarabine-like chemotherapy for 7 days and anthracycline-like chemotherapy for 3 days. No further therapy is applied and the course of the leukemia is observed until the end of the simulation (2000 days, 5000 days for the slow pace leukemia).

A variation of the 7 + 3 chemotherapy regimen that is based on the 2017 guidelines of the National Comprehensive Cancer Network (NCCN) [1] has also been implemented. Analogously to classical 7 + 3 the prescribed combination therapy will be applied, if blast fraction exceeds 20%. A first evaluation of therapy success by assessing blast fraction is made on day 14 after treatment start. A second induction therapy will be performed, if blast fraction ≥5.5%. However, second induction therapy will be intensity-reduced (5 days of cytarabine-like chemotherapy and 2 days of anthracycline-like chemotherapy (5 + 2)). If blast fraction is < 5.5%, a second evaluation will be made on day 21 after treatment start. In that case, a blast fraction ≥5% also leads to a second therapy course (5 + 2). For blast fractions < 5% no further treatment will be applied. Within one simulation run intensity values (*k*_*cyt*_, *k*_*anthra*_) will be preset and fixed for 7 + 3 and 5 + 2.

To identify a realistic range of chemotherapy intensities (*k*_*cyt*_ and *k*_*anthra*_, in unit cell deaths per day) we sampled therapy combinations starting with no therapy (*k*_*cyt*_= *k*_*anthra*_=0) and increased intensities until we reached the area of overtreatment (i.e. no complete remission can be achieved and all compartments are completely depleted by intensive therapy). By intention, we included the mono-treatment scenarios. While cytarabine mono-treatment is common practice in the pre-phase, consolidation and treatment of the elderly [1] anthracycline mono-treatment is uncommon in clinical practice. Finally, we simulated all scenarios between intensity values between 0 and 10 (step size 0.1). We could show for the selected fast and intermediate leukemia that for *k*_*cyt*_>8.8 (mono-therapy) in the standard arm and *k*_*cyt*_> 5.8 in the evaluation regimen leads, regardless of *k*_*anthra*_, leads to overtreatment (complete depletion of all compartments). Under-treatment exists for low *k*_*cyt*_and *k*_*anthra*_ rates that lead to no CR. As internal validation, we also measured absolute reduction of leukemic cells (l_1_ and l_2_) per chemotherapy combination on day 29 after treatment start (compared to leukemic cell abundance at diagnosis). For the effective regions (no under/over-treatment) we observed reductions between 10^5^and 10^9^ cells, resembling realistic values [39].

**Fig. 2:**
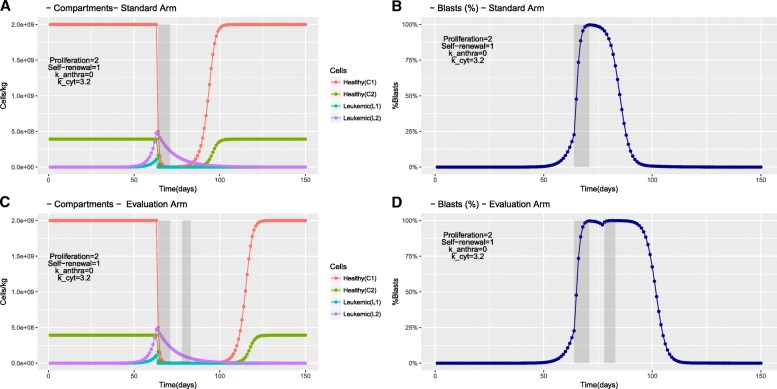
Exemplary simulations of a fast pace leukemia. The simulations started in the steady state of the healthy hematopoietic model. A small amount of leukemic cells (l_1_) with proliferation *p* = 2 and self-renewal a = 1 was introduced at Time = 0. The simulation shows the development of a leukemia that reaches the diagnostic threshold (20% blasts) at day 63. Treatment starts immediately (monotherapy with 7 days cytarabine). Healthy and leukemic compartments immediately react to the chemotherapy and get depleted. Panels **a** and **b** show the simulated trajectories within the standard arm. The neutropenic phase lasts ca. 20 days (Panel **a**). During this phase the relative blasts count (%, panel **b**) is high due to the very low absolute cell counts. This simulation shows the occurrence of a complete remission. The l_1_ cells (HSC) can be cleaned by therapy. The total blasts clearance (also for the l_2_ compartment) takes until day 90 to 100. Panels **c** and **d** (Evaluation arm) show, in contrast to the standard arm a prolonged neutropenic phase (plus additional 20 days). This is induced by the second course, which is applied during the already recovering normal hematopoiesis

An exemplary simulation of a fast leukemia under a specific treatment dose is shown in Fig. 2, which depicts the cell number trajectories and blast percentages over time.

### Outcomes

To characterize the three scenarios (fast, intermediate, and slow) we derived the following outcomes:

- Time to complete remission (CR): Complete remission: blast fraction ≤5% [5]. We measured time in days from diagnosis to first time CR.
- Duration of CR: In case of a CR, we measured time from CR to relapse (blast fraction > 5%) or respectively until all HSC are depleted. In our simulations we used a threshold that set cell counts (c_1_, c_2_, l_1_, l_2_) to zero if they are respectively below one cell (here, < 1/70). We consider such a situation as complete loss of normal hematopoiesis that very probable leads to death. If none occurs (neither relapse nor loss of normal hematopoiesis), the time from CR until the end of the simulation run was reported (2000 days, 5000 days for the slow pace leukemia).
- Therapeutic width: Therapy combinations will vary in their effectiveness. We thus also report the relative frequency of therapy combinations leading to a CR (therapeutic width), as well as the relative frequency of therapy combinations resulting in under- and overtreatment, respectively.

Resulting outcomes will be presented as heatmap figures in the manuscript. Outcome data is contained in Additional file 3.

## Results

### Time to diagnosis (pre-treatment phase)

**Fig. 3:**
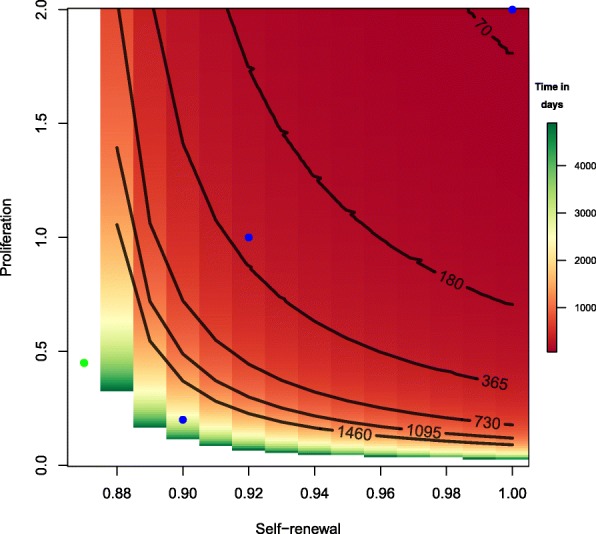
Time to 20% blasts. We simulate all combinations of leukemic proliferation (*p*^*l*^, range: 0–2) and self-renewal (*a*^*l*^_max_, range: 0–1) rates. Each parameter combination can be considered a different leukemia. We observed the time to reach the diagnostic threshold (20% blasts). Black lines indicate exemplary contour lines (parameter combinations leading to the same time to diagnosis). The plot shows that leukemia only occurs when the leukemic cells outcompete the healthy cells. The green filled circle indicates the parameter combination of the healthy HSCs. No leukemia occurs for parameter combinations with self-renewal < 0.87 (healthy self-renewal). A reduced proliferation can lead to a leukemia in combination with increased self-renewal. Simulations were run for 5000 days (13.7 years), but leukemic clones that resides within a patient for such a prolonged subclinical phase might not be considered an acute leukemia anymore. Three parameter combinations (fast, intermediate and slow pace; blue filled circles, Table 2) were selected for further analysis

Figure 3 shows the results for different parameter combinations for self-renewal (*a*^*l*^) and proliferation (*p*^*l*^). Fast and high self-renewal leukemia can occur in a very short time (60 days). Slower leukemias can persist on low, undetectable levels, for several years before a detectable blast count is produced. Leukemic stem cells (LSC) always need an increased self-renewal rate to outcompete HSC. A reduced proliferation rate can also lead to a diagnosable disease if self-renewal is increased. This finding is in accordance with prescribed similar findings [16, 40]. We used this information to select three parameter combinations (slow, intermediate, fast leukemia; Fig. 3 and Table 2).

**Table 2:** Parameter values of the three analyzed parameter combinations

Pace	Self-renewal	Proliferation	Time to diagnosis
slow	0.9	0.2	2756 days
intermediate	0.92	1	316 days
fast	1	2	63 days
healthy HSC^a^	0.87	0.42	–

^a^Parametrization of the healthy system according to [13]

We selected three leukemias representing three different paces (fast, intermediate, slow) using the time to 20% blasts (diagnostic threshold) as criterion. Full model parametrization is given in Additional file 1.

Before presenting treatment results we emphasize that we used the same “standard patient parameters” for every simulation [see Additional file 1]. As a result, percentages of CRs cannot be compared directly to known clinical CR rates, which are generated from a patient population. Additionally, we restrict the AML treatment to the induction therapy, in order that we can analyze the isolated effect of induction treatment.

### Slow-pace leukemia

Our simulations show that the selected slow-pace leukemia cannot be treated to CR. Nevertheless, we can record a significant reduction of leukemic cells on day 29 after treatment start (at that time in our simulation a potential second induction cycle has been administered). Reduction in absolute cell numbers ranges from about 10^5^ to 10^9^ for standard regimen and from about 10^4^ to 10^9^ for evaluation regimen. However, for CR (leukemic cells under 5%) required intensities lead inevitably to complete depletion of healthy cell lines.

### Fast-pace leukemia

**Fig. 4:**
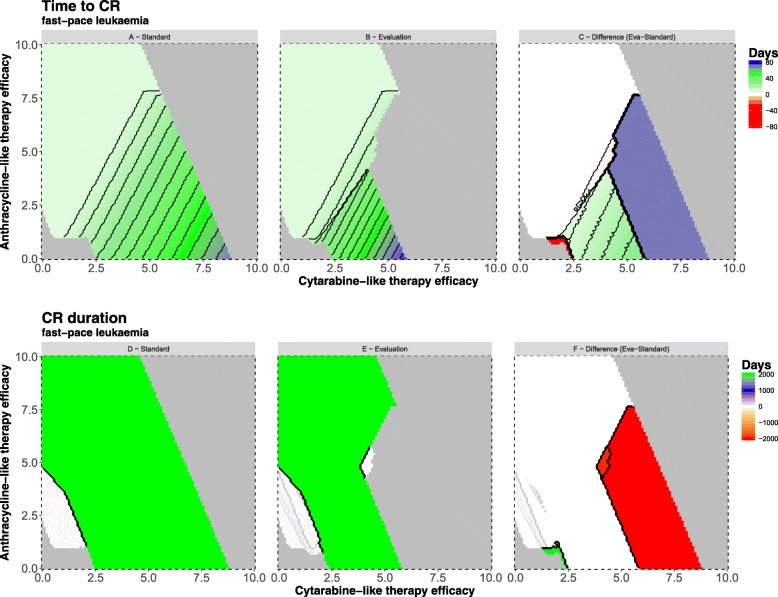
Time to complete remission and duration of complete remission for a fast-pace leukemia. For the selected fast-pace leukemia we simulated all therapy-intensity combinations for the 7 + 3 combination therapy (range: 0–10) for both study arms. **a** and **b** show the days from diagnosis to CR. **c** shows the difference “Evaluation – Standard”. The gray areas show treatment intensities which lead to no CR (lower left area because of under- and upper right area because of overtreatment). The black lines represent selected contour lines (i.e.treatment intensities with same values). Considering a shorter time to CR as beneficial, a difference > 0 indicates a benefit for the standard arm. Differences < 0 indicate that the evaluation regimen is beneficial. Blue and red regions in the plot indicate where one of the two regimens allows for CR while the other does not, respectively. In particular, the blue area covers treatment combinations where the standard treatment leads to CR while the evaluation regimen already results in overtreatment. The red area shows that the evaluation regimen can be useful for low dose treatments. Here the evaluation and the second course allows to reach a CR where the standard regimen results in undertreatment. Plots **d**, **e**, and **f** display the CR duration. Due to the setup of the simulation no relapses occur thus simulation time after CR onset is considered. The difference between evaluation and standard shows two regions (red) where standard leads to longer CR durations. The green region shows where evaluation regimen allows for a CR while the standard arm does not

Figure 4a shows the resulting time to CR values for each simulated therapy combination under standard regimen. In general, we can observe that a region with effective therapies exists and is flanked by a region (lower left) we denote “undertreatment” and “overtreatment” region (upper right). Overtreatment decreases the abundance of healthy HSC faster than the LSC and no CR is achieved. Compared to clinical practice, this effect could be analogous to chemotherapy toxicity leading to patient’s death. Low chemotherapy is not able to reduce leukemic burden in bone marrow effectively and no CR is reached, either.

Higher cytarabine-like intensities lead to prolonged times to reach a CR. This effect is due to the simultaneous reduction of LSC and HSC. Time to CR will be shortened if anthracycline-like intensities increase, i.e. additional decrease of the differentiated cells.

In total, 64.28% of simulated intensity combinations result in a CR (Fig. 4a, Table 3). Between 8 and 70 days can be necessary to achieve CR. An early CR, at day 8, is reached mostly by increasing the effectiveness of anthracycline-like therapies. A cytarabine-like monotherapy has a minimal time to CR of ca. 25 days. A large fraction of therapy combinations (45.75%) lead to an early CR (within 8 days).

**Table 3:** Outcome parameters for fast and intermediate paced leukemias

	Standard arm	Evaluation arm
Fast-pace leukemia		
Time to CR (range)	8 to 70 days	8 to 78 days
Therapeutic width^a^	64.28%	46.39%
Overtreatment region^a^	33.03%	51.24%
Undertreatment region^a^	2.70%	2.37%
Intermediate-pace leukemia		
Time to CR (range)	8 to 69 days	8 to 78 days
Therapeutic width^a^	63.65%	46.70%
Overtreatment region^a^	33.55%	51.16%
Undertreatment region^a^	2.74%	2.12%

^a^ % combinations

In the evaluation regimen 46.39% of simulated intensity combinations lead to a CR (Fig. 4b, Table 3). The range of required days to CR is between 8 and 78 days. As before, an early CR is mostly due to increasing anthracycline-like effectiveness. Minimal time to CR under cytarabine-like monotherapy accounts for approx. 35 days. Fastest possible CR (on day 8) can be reached in 63.40% of CR combinations.

Due to the second induction therapy course, overtreatment is more prominent in the evaluation regimen (Evaluation: 51.24% vs. Standard: 33.03%). Undertreatment is slightly less likely (Evaluation: 2.37% vs. Standard: 2.7%).

Figure 4c shows that 71.27% of combinations do not differ in time to CR. Reasons are (i) that the additional evaluation does not lead to further treatment (sufficient blast clearance) and (ii) overlapping under- and overtreatment regions. Comparing the minimal required effective anthracycline-like monotherapy shows no difference. Minimal required effectiveness of cytarabine-like monotherapy is slightly decreased under evaluation regimen (*k*_*cyt*_=2.4 vs *k*_*cyt*_=2.5).

Single-induction 7 + 3 outperforms the evaluation regimen in 27.36% of combinations (Fig. 4c, blue region). The evaluation regimen leads to a CR in 1.36% of combinations, where standard regimen does not allow for a CR (Fig. 4c, red region). These are therapy combinations, which are considered “undertreatment” in 7 + 3. The evaluation regimen can be applied with lower intensities. Other therapy combinations do not show a clear advantage or, worse, might even prevent faster recovery of normal hematopoiesis (elongation between 0 and approx. 20–30 days).

Figure 4d and e show the CR duration. Two groups can be identified. On the one hand a very short and on the other hand a prolonged CR. In the model LSC are completely cleared, leading to quasi-infinite CRs. Short-time CRs range between 1 and 11 days. Quasi-infinite CRs account for 59.79% and short CR for 4.49% of all simulated combinations.

Increasing anthracycline-like monotherapy effectiveness results initially in very short CR under lower effectiveness and subsequently with higher level in quasi-infinite CR. On the other hand, cytarabine-like monotherapy leads directly to lengthy CR at a certain level of effectiveness.

In the evaluation regimen, short and quasi-infinite CR represent of 5.17 and 41.22% of all combinations, respectively. Range of short CR is between 1 and 18 days.

69.27% of all treatment combinations do not differ in CR duration. Standard regimen achieves longer CR durations in 28.89% of combinations (Fig. 4f, red region). The evaluation regimen only in 1.84% of combinations. However, under the evaluation regimen longer lasting CR are established by lower chemotherapy intensities (Fig. 4f, green region). For the cytarabine-like monotherapy the evaluation regimen shifts the minimal effectiveness from k = 2.5 to k = 2.4. Figure 4f show that a further reduction in cytarabine-like effectiveness can only be achieved by increasing the anthracycline-like effectiveness. An increase in anthracycline-like effectiveness to k = 1.2 allows to reduce cytarabine-like effectiveness to k = 1.9 while still maintaining a quasi-infinite CR. All other quasi-infinite CRs can only be reached with either higher cytarabine-like or anthracycline-like effectiveness, e.g. by increasing chemotherapy doses. Evaluation regimen prevents the occurrence of quasi-infinite CR (or leads only to very short CRs) in a large number of therapy combinations, where standard 7 + 3 leads to a quasi-infinite CRs (Fig. 4f, red region).

### Intermediate-pace leukemia

**Fig. 5:**
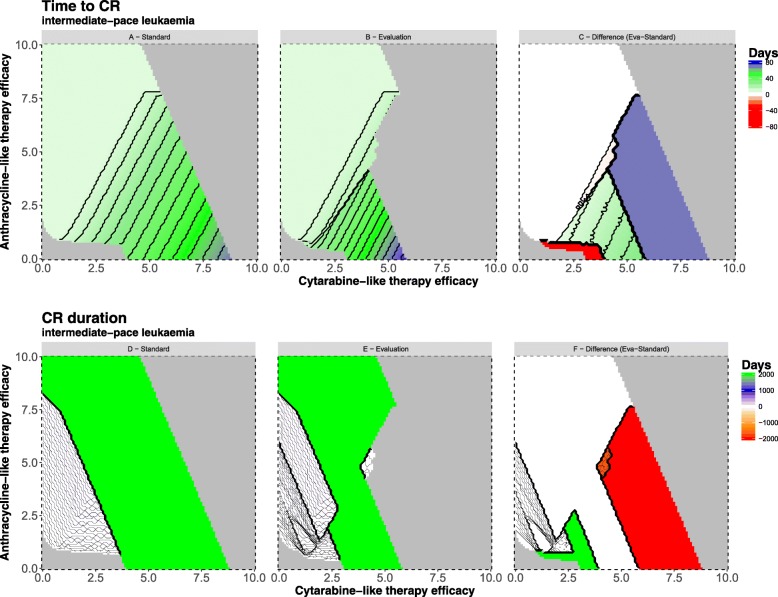
Time to complete remission and duration of complete remission for an intermediate-pace leukemia. The intermediate paced leukemia shows qualitatively the same results as the fast paced leukemia (Fig. 4). The pace type shifts the regions suggesting how treatment efficacy affects therapy success. The color-coding is described in the caption of Fig. 4

Regarding the intermediate pace leukemia under standard regimen, we observe that 63.65% of computed therapy combinations result in a CR (Fig. 5a, Table 3). Required times to CR range between 8 and 69 days. A cytarabine-like monotherapy shows a minimal time to CR of ca. 35 days. The minimal required effectiveness of anthracycline-like monotherapy leading to a CR is lower than in the fast pace leukemia (*k*_*anthra*_ =2.3, fast paced leukemia; *k*_*anthra*_=1.8, intermediate pace leukemia). The required effectiveness of cytarabine-like monotherapy resulting in a CR is larger (*k*_*cyt*_=2.5, fast paced leukemia; *k*_*cyt*_ =4.0, intermediate pace leukemia). Quickest possible CR on day 8 forms again the largest group among all therapy combinations leading to CR with 46.76%. Comparing potential overtreatment combinations there is no difference between the intermediate and the fast pace leukemia under standard regimen (Table 3). Our model shows that more chemotherapy combinations fail to reduce leukemic burden effectively for the intermediate pace leukemia in contrast to the fast pace leukemia under standard regimen (2.70% (fast) vs. 2.74% combinations (intermediate)).

For the intermediate pace leukemia under evaluation regimen 46.70% of intensity combinations result in a CR (Fig. 5b, Table 3). Range of time to CR is between 8 and 78 days. Minimal duration to CR under cytarabine-like monotherapy is ~ 45 days. Minimal required effectiveness of anthracycline-like monotherapy leading to an early CR is lower for the intermediate pace leukemia (*k*_*anthra*_ =2.3, fast pace leukemia; *k*_*anthra*_ =1.8, intermediate pace leukemia). Required effectiveness of cytarabine-like monotherapy resulting in a CR is increased (*k*_*cyt*_ =2.4, fast pace leukemia; *k*_*cyt*_ =3.1, intermediate pace leukemia). Quickest possible CR on day 8 represents again a major portion (63.73%) of all CR combinations.

Under evaluation regimen our model shows that more chemotherapy combinations fail to reduce leukemic burden effectively for the fast pace leukemia in contrast to the intermediate (2.12% (intermediate) vs 2.37% combinations (fast)) (Fig. 4b and 5b, Table 3). Comparing standard and evaluation regimen for the intermediate pace leukemia, we can observe more treatment combinations result in undertreatment in the standard regimen (2.74% (standard) vs 2.12% (evaluation)) and more combinations of overtreatment under evaluation (33.55% (standard) vs. 51.16% (evaluation)).

Regarding absolute difference between standard and evaluation regimen 71.53% of simulated combinations do not differ in time to onset of complete remission (either same time or no CR is achieved: 36.37% vs 35.16%, Fig. 5c). A faster CR (alternatively in the first place a CR) is achieved in 26.26% of simulated combinations under standard regimen. Evaluation regimen only allows this superior scenario in 2.21% of cases (Fig. 5c, red region). The evaluation regimen can provide more therapy combinations resulting in a CR by lower chemotherapy intensity compared to standard regimen.

Furthermore, we can record that (as with the fast pace leukemia) minimal required effectiveness of anthracycline-like monotherapy is the same under standard and evaluation regimen (*k*_*anthra*_=1.8). Required effectiveness of cytarabine-like monotherapy resulting in CR is lower under evaluation regimen (*k*_*cyt*_=3.1) compared to 7 + 3 single induction (*k*_*cyt*_=4.0).

The intermediate pace leukemia under standard regimen shows again very short CR durations (15.05%) and quasi-infinite CR durations (48.06%) (Fig. 5d). Short CRs range from 1 to 25 days. As with the fast pace leukemia total lengthy CR under standard treatment can be considered as persistent lasting until the end of simulation (2000 days).

Within the evaluation regimen, higher cytarabine-like therapy intensities also exhibit short CR durations (Fig. 5e). 14.67% of all simulated therapy combinations are short CRs while 32.03% are prolonged CR.

66.81% of all combinations do not differ in duration of CR between standard and evaluation regimens (Fig. 5f). Standard regimen provides longer lasting CR (25.71%) compared to evaluation regimen (7.48%). Under evaluation regimen lengthier CR require lower chemotherapy intensity to achieve CR.

## Discussion

We modelled three leukemias by varying proliferation and self-renewal rates leading from initial mutation to three different times of diagnosis. Results from AML pathogenesis research show that growth properties (such as proliferation rate) affect different survival outcomes [41, 42]. This finding is also supported by mathematical modeling results [40]. Quantifying leukemias by growth kinetics (e.g. leukemic pace as time-to-diagnosis) is relevant. Various mutations were identified and specific cytogenetics are linked to different patient outcomes [43–45]. However, there is no information about the time to diagnosis for specific leukemia types and how chemotherapy influences dynamics of hematopoiesis over time. For example, bone marrow examinations are performed for diagnosis and 7 to 10 days after induction chemotherapy [5]. In between, no continuous data is collected to reduce patient burden. When modern cytometric techniques get more available this gap can be closed. These procedures, especially if based on bone marrow samples, cannot be used for continuous monitoring [46]. Mathematical models are able to bridge this diagnostic gap and can reveal suspected useful therapeutic implications by its dynamical approach.

Here, we presented results based on a homogenous approach (one type of leukemic cells) to characterize the dynamic behavior of certain AML subtypes. Nevertheless, it is known that AML is a multiclonal disease [4, 35]. We modelled a combination chemotherapy attacking this one leukemia and summarized the simulated therapy combinations. In reality, a mixture of leukemic cells with different properties is observed at diagnosis and a clonal evolution leading to relapse can be demonstrated [4, 47]. Despite of this variety, in most cases a dominant clone induces AML onset [48]. We only focused on induction therapy’s impact on this dominant clone. Post-remission therapy like consolidation chemotherapy is intentionally not considered and is subject of future research. As a consequence, our comparison of two induction regimens aims exclusively at an improvement of CR achievement without considering effects on potential relapse in the course of clonal evolution. Our presented results must be assessed in the specific model context and a direct comparison with usual clinical outcome or endpoint parameters, which are based on patient populations, cannot be made instantly. In our focused consideration, relapses by different clones are not possible, in order that quasi-infinite CRs are obtained by induction treatment by complete depletion of a single leukemic clone. In multiclonal models relapses are expected.

With respect to realistic therapy concepts several limitations occur. The leukemia’s sensibility to chemotherapy usually is influenced by drug resistance mechanisms [49, 50]. The therapy effectiveness (*k*_*cyt*_, *k*_*anthra*_) can be considered as combinations of therapy intensity (dose) and the leukemia’s resistance (only affected by proliferation rate and cell numbers) to the therapy, e.g. due to its specific genetic characteristics. At present, the model cannot simulate an AML-type specific resistance. Future model extensions will aim for data-derived proliferation and self-renewal parameters (representing specific genotypes) and respective resistance mechanisms. Besides, in clinical practice, chemotherapy intensity is applied in units of *mg*/*m*^2^ adapted to body surface to take account of side effects [5]. Currently, we cannot compare model parameters to clinical therapy intensities directly. The model is at least able to review different doses qualitatively on a course-grained scale (high vs low). Another future prospect will be to link the model parameters in a pharmacodynamics model to therapy doses, to e.g. replay studies intensifying induction by dose increase [51–53]. With respect to the selected leukemia parameter combinations via leukemic pace our selection might bias our conclusions if leukemic proliferation and self-renewal parameters influences our selected outcome measures significantly. Additional simulations (data not shown) indicate that time to CR is not influenced significantly by leukemia parameters. For lower intensity therapy combinations CR duration is related to the leukemias proliferation rate, while self-renewal has no effect. There seems to exist a threshold proliferation rate. Below this threshold, only very short CRs can be observed. While we focused on therapy intensity in this publication, a more elaborate analysis of the interplay between leukemia characteristics and therapy outcomes will be necessary and should be investigated in future research.

To assess the modelled chemotherapy values compared to realistic used intensities, we used the established criterion of 3 log_10_ cytoreduction, which is at the minimum required for a reduction of leukemic cells under 5% in bone marrow [39]. In addition, a reduction of transcription products of more than 3 log_10_ levels is also used as a prognostic factor in minimal residual disease (MRD) monitoring after induction therapy [54–56]. Therefore, in relation to an adequate chemotherapy intensity a log_10_ reduction ≥ 3 of leukemic cells can be considered as a predictor for treatment success. All chemotherapy intensities leading to CR feature a leukemic cell reduction > 3 log_10_ levels. In fact, in the model reductions often exceed this criterion. Referring to minimal detection level of minimal residual disease (MRD) with sensitivities between 10^− 4^ and 10^− 5^ [46], our model provides a starting basis for further and novel MRD investigations by demonstrating cell trajectories (with precise blast percentages) over time (Fig. 2). Common medical diagnostics cannot enable a comparable continuous view.

In the next steps, patient data like healthy steady state stem and progenitor cell numbers must be integrated into the model. In future, exact cell number analysis of a patient will be challenging, especially the knowledge transfer from mice models to manageable in-vivo analysis [43]. At the same time, a determination of self-renewal must be derived from this patient individual data [13, 45]. Availability of personalized parameter values lead to a further specialized model would be able to translate full spectrum of genetic change into specific values of proliferation and self-renewal [57]. Subsequently, each individual AML as an own genetic entity and effects of therapy could be highly effectively modelled and assessed. Regarding this, the categorization of AML types by current classification systems (e.g. ELN, MRC, WHO, FAB) considering cytomorphological, genetical and immunological properties is complex because of known heterogeneity of AML [5]. These classifications especially regard rather static properties like mutations or the immunophenotype. Derived aggregations lead to risk groups that comprises similar patient outcomes, but they do not precisely describe how fast the AML proliferates, nor which resistance mechanisms exist and which consecutive dynamical impact on the hematopoietic system is generated. Hereby, the mathematical model provides a functional perspective, which allows a more individual analysis of the AML pathogenesis and therapy effects.

Varying therapy regimens are used worldwide, that mostly differ in time and duration of chemotherapy administering [8]. The presented treatment model is also suitable for a planned comparison of different double induction concepts like TAD-HAM vs. S-HAM [7]. In addition to our evaluation regimen investigation, we will analyze different evaluation time points to find out, when bone marrow assessment should be optimally done. Indeed, evaluation timing is still an open issue and our dynamical model could be helpful to provide additional valuable insights [58–60].

Risk-stratification concepts for AML therapy are currently gaining in importance and are particularly established in post remission therapy [61–63]. Hereby, treatment strategy is especially influenced by estimated outcome [64]. We could observe that personalized treatment intensities in induction chemotherapy produce relevant advantages (lower minimum necessary chemotherapy intensities). Therefore, prospective stratification concepts might also include inherent properties of AML.

## Conclusions

Complementary to randomized controlled trials (RCT), modelling can be understood as a tool that can add a holistic point of view to classical reductionist medicine [65]. Moreover, clinical relevant modelling consists of a hypothesis-driven research, that connects *in-silico* experimental results with established experimental facts in a scientific interacting cycle [66, 67]. Concerning an effective personalized medicine in AML treatment, we are convinced that this interdisciplinary approach will be inevitable and offers great potential. At present, our model can derive clinical relevant conclusions despite the prescribed limitations, because our integral dynamical approach enables new insights in AML-hematopoiesis and optimal chemotherapy effect relating to a certain AML types.

Our results suggest, that the “7 + 3” regimen results in CR more often. In addition, more therapy combinations result in quasi-infinite CR. This holds for the fast and the intermediate paced leukemia (in the model the slow paced leukemia is not treatable to achieve CR). The results support the current scientific view that “7 + 3” regimen is a standard of care independent from existing diverse regimen variations that are applied in study groups all over the world [7]. Nevertheless, a more extensive evaluation and comparison with many more established therapy schemata is necessary.

We assume that genetic heterogeneity of every leukemic clone determines unique characteristics that require corresponding unique therapy concepts. This assumption is based on significantly different survival outcomes that are strictly dependent on specific genetic constitution [5]. Induction therapy is not routinely adapted to genetic disposition and patients are treated with standardized induction doses only adapted to body surface [5]. Concepts of higher doses or adding a third agent were implemented in several randomized studies, but the comparison proved difficult and dose increases were not precisely adapted to individual patient [7]. Regarding this, our model suggests that a whole spectrum of effective (that means CR as well as prolonged CR) chemotherapy intensities from relatively low to high exists and “7 + 3” regimen offers a larger effective spectrum that is respectively adjusted to characteristics of considered clone type. On the hypothesis that these insights hold true concerning real life, “7 + 3” regimen could be lead to higher cure probability of a standardized dose applied to heterogeneous diseases because of more existing effective dose combinations for each AML type. Nevertheless, a more substantial model based comparison of “7 + 3” and other therapy regimens is lacking.

In our simplifying model, the essential criterion for an optimal clinical outcome is ultimately duration of CR. As a result, we initially consider different intensity combinations as equivalent, as far as achieved CR duration is similar. However, side effects of chemotherapy are modelled via a cytotoxic effect on blood cells (indeed model is calibrated to number of neutrophil granulocytes as most frequent leukocytes [13]). Thus, lower and higher intensities leading to same CR duration only differ in absolute cytoreduction without affecting the defined outcome. Other significant side effects concerning for example the gastrointestinal tract or residual blood system (or anyway related infections) are not factored in. For that reason, we consider lower intensities leading to same CR duration as superior. There is growing evidence that a relevant part of progress in AML outcome is due to improved supportive therapy [68–70]. Finding lowest as possible and at the same time effective therapy intensities seems to be eminently important [71]. To that effect, we observe for both regimens that spectrum of effective therapy intensities leading to CR as well as persistent CR only differs in the region of lowest intensities. We can derive from this model results that lower and still effective induction therapy intensities may exist depending on different AML clone type. In the concrete case of our model, a fast leukemia can be treated efficiently with lower intensities than the intermediate leukemia. We record that the evaluation regimen enables lowest intensities leading to prolonged CR (i.e. most efficient treatment) for both selected parameter combinations.

It should be noted that evaluation regimen provides these lower effective intensities especially for the intermediate paced leukemia, because hereof largest reduction of essential intensity (in relative comparison to “7 + 3”) is obtained. We emphasize that in our model evaluation regimen does not offer more effective therapy combinations but the most efficient regarding optimal outcome and respective minimum intensity. Hereby, in our model the evaluation approach is particularly worthwhile to minimize therapy intensities and consecutive side effects with regard to medium-fast proliferating leukemia. The main potential of the evaluation approach is presumably present for AML of poorer risk categories. This insight complies inherently with current results that discusses necessity of bone marrow assessment and recommends a more individualized decision of evaluation [72].

An early response to the first induction cycle is a known prognostic factor, but its impact on evaluation process stays unclear [58, 73]. Our results show that therapy combinations enabling fastest CR (in our model quasi-instantly after chemotherapy) constitute the majority of achievable CR independent of administered regimen and leukemia pace. In consideration of intensity and optimal outcome, we detected therapy combinations leading to CR within 20 to 45 days. Concerning this efficient subset, in general, “7 + 3” enables faster CR and in addition, the intermedium paced leukemia takes longer time to CR. Persistent CR under minimal dose (the most efficient situation) is obtained by evaluation regimen regardless of whether other therapy combinations lead to even faster CR.

Published literature proposes that CR should be reached as fast as possible [74–76]. According to our modeling results, therapy combinations leading to CR within the first 10 days does not always enable a long-lasting CR. A therapy intensification can lead to longer times until onset of CR, but then lead also to a more stable CR. In summary, in our model fast as possible CR achievement is not inevitably optimal.

Enabling persistent CR with examined minimal dose, cytarabine-like monotherapy turns out to be optimal for both regimens. This model result conflicts with clinical reality, which ascribes relevant importance to a combination chemotherapy for decades [38]. Our model considered one type of leukemic cells per patient (see model limitations above). We know from recent studies, that in one patient several subtypes exist and combination chemotherapy leads to a selection process [13, 47, 57]. Some subtypes are more resistant to this chemotherapy for several reasons (e.g. a lower proliferation rate). In our homogenous model, we do not examine the prescribed selection process, because we focus on the treatment effect regarding one specific AML. The success of classical “7 + 3” cannot be considered as resounding because of existing numerous AML subgroups with poor survival outcomes [5]. Therefore, multi-layered approaches of targeted therapy (e.g. immunotherapy or pathway inhibition) are recently under investigation without major breakthrough until now [7].

## Additional files

Additional file 1: Extended description of the mathematical model including the full parametrization. (DOCX 30 kb)

Additional file 2: R script file containing the program to simulate the AML model. (R 7 kb)

Additional file 3: File containing the data that was used to generate Figs. 3, 4 and 5. (TXT 3809 kb)

## Abbreviations

AML: Acute myeloid leukemia
BM: Bone marrow
CR: Complete remission
ELN: European Leukemia network
FAB: French – American –British Classification
HSC: (Healthy) Hematopoietic stem cells
LSC: Leukemic stem cells
MRC: Medical Research Council
MRD: Minimal residual disease
RCT: Randomized Controlled Trial
TAD-HAM, S-HAM: Combination chemotherapy schemata
WHO: World Health Organization
