# Supplementary material for: In-silico comparison of two induction regimens (7 + 3 vs 7 + 3 plus additional bone marrow evaluation) in acute myeloid leukemia treatment
Source: BMC Syst Biol. 2019 Jan 31;13:18. doi: 10.1186/s12918-019-0684-0 (PMC6357450; doi:10.1186/s12918-019-0684-0)
Supplement: Supplementary file 1 — Extended description of the mathematical model including the full parametrization. (DOCX 30 kb) [file 12918_2019_684_MOESM1_ESM.docx]

**Additional File 1 – Mathematical Model**

Article:
In-silico comparison of two induction regimens (7+3 vs 7+3 plus additional bone marrow evaluation) in acute myeloid leukaemia treatment

Jan Christoph Banck^1,2^, Dennis Görlich^1^

^1^Institute of Biostatistics and Clinical Research, Westfälische Wilhelms-Universität Münster

^2^ IIIrd Department of Medicine, University Hospital Großhadern, Munich, Germany

For our analysis, we used and extended the published AML model by Stiehl et al. [1].

Within the model normal haematopoiesis is represented as of two compartments, i.e. haematopoietic stem cells (HSC) within the bone marrow which can differentiate by mitotic cell division into non-proliferating (differentiated) cells. The simplification of a two-compartment model allows to concentrate on the general properties of haematopoiesis.  Thus, the model is able to explain the dynamics of cell population abundance adequately [1,2].

We also assume that leukemic stem cells (LSC) also can be modelled in two compartments. Nevertheless, they exhibit different characteristics that change their dynamic behaviour. Also, the interpretation of the second compartment is different compared to normal haematopoiesis, i.e. LSC “differentiate” incompletely into blasts.

Each cell type (i.e. normal HSC and pathological LSC) will be represented by a set of two ordinary differential equations. Abundance (in cells/kg body weight) of a compartment at time t (in days) is denoted by $c_{1}\left( t \right)$for HSC, $c_{2}\left( t \right)$for healthy differentiated cells, $l_{1}\left( t \right)$for LSC and $l_{2}\left( t \right)$for non-proliferating leukemic cells. As part of the mathematical model HSC, LSC and non-proliferating leukemic cells are located in the bone marrow, whereas healthy differentiated cells belong to the blood stream. Effects of this distinction are explained later.

Hereof, in the model the proliferation rates $p^{c}$ (HSC) and $p^{l}$ (LSC) describe how often stem cells divide per day. In particular, in the process of cell division one fraction of daughter cells maintain their ability to divide (self-renewal) and the other fraction differentiate to non-mitotic cells. In the model the term of self-renewal ($a^{c}$ for HSC, $a^{l}$ for LSC) represent this self-sustaining fraction as a proportion (0-1). Proliferation rates between 0 and 2 and self-renewal rates between 0 and 1 are considered.

The following system of ordinary differential equations describes the rate of change for each cell type:

$\frac{d}{dt}c_{1}\left( t \right)=2\cdot a^{c}\cdot p^{c}\cdot c_{1}\left( t \right)-p^{c}{\cdot c}_{1}\left( t \right)$ $\left( 1 \right)$

$\frac{d}{dt}c_{2}\left( t \right)=2{\cdot p}^{c}\cdot c_{1}\left( t \right)-{2a^{c}\cdot p}^{c}\cdot c_{1}\left( t \right)$ $\left( 2 \right)$

$\frac{d}{dt}l_{1}\left( t \right)=2\cdot a^{l}\cdot p^{l}\cdot l_{1}\left( t \right)-p^{l}\cdot l_{1}\left( t \right)$ $\left( 3 \right)$

$\frac{d}{dt}l_{2}\left( t \right)=2{\cdot p}^{l}\cdot l_{1}\left( t \right)-{2{\cdot a}^{l}\cdot p}^{l}\cdot l_{1}\left( t \right)$ $\left( 4 \right)$

In $\left( 1 \right)$ the minuend involves absolute number of HSC at time t after multiplication by cell division, considering effects of self-renewal. The subtrahend regards pre-existing number of HSC, so that the entire term $\left( 1 \right)$ describes the absolute increment of HSC (in cells/kg body weight) at time t.

The minuend in $\left( 2 \right)$ covers the multiplication of all HSC per day without considering effects of self-renewal. Therefore, the subtrahend comprises number of HSC after multiplication, which maintain their ability of proliferation. As a result, the subtraction term $\left( 2 \right)$ describes the absolute increment of healthy non-proliferating cells (in cells/kg body weight at time t).

Accordingly, $\left( 3 \right)$ comprises the increase of LSC and $\left( 4 \right)$ the increase of leukemic blasts at time t.

In previous work this basic model of growth was expanded by specific features to image appropriately the biological process of normal plus pathological haematopoiesis [3,4]. In addition, the impact of a chemotherapy was examined as well [1].

Within the scope of these papers a feedback regulation denoted by$s\left( t \right)$ was implemented to represent cellular communication. Affecting proliferation and self-renewal, feedback regulation leads to the result that increasing number of differentiated healthy cells causes a reduced number of HSC and LSC (and vice versa). In the considered specific model leukemic cells are independent of this feedback regulation.

Additionally, constant death rates of non-proliferating cells ($d_{2}^{c}$ and $d_{2}^{l}$) describe, which fraction of these cells dies per time t. In the model, mitotic cells do not die on a regular basis.

Physical space in bone marrow is limited. Therefore, there resident cells (here $c_{1}$, $l_{1}$ and $l_{2}$) will die, if bone marrow cell number exceed a certain threshold value. Function $d\left( x_{(t)} \right)$ takes account of this threshold dependent on the physiological equilibrium value of bone marrow cell count. Similar to the constant death rates, $d\left( x_{(t)} \right)$works as an additional death rate that describes the fraction of bone marrow cells dies because of overcrowding.

Furthermore, a mono-chemotherapy acting proportionally on proliferating cells was introduced (denoted by a constant factor $k_{chemo}$) [1]. In this way, effectiveness of chemotherapy is the higher, the more cell divisions occur.

The following mode combines a mono-therapy and the pathological dynamics:

$\frac{d}{dt}c_{1}\left( t \right)=2\cdot a^{c}\cdot p^{c}\cdot{s\left( t \right)\cdot c}_{1}\left( t \right)-p^{c}\cdot c_{1}\left( t \right)-d\left( x_{(t)} \right)\cdot c_{1}\left( t \right)-k_{chemo}\cdot p^{c}\cdot c_{1}\left( t \right)$ $\left( 5 \right)$

$\frac{d}{dt}c_{2}\left( t \right)=2\cdot p^{c}\cdot c_{1}\left( t \right)-{2\cdot a^{c}\cdot p}^{c}{\cdot s\left( t \right)\cdot c}_{1}\left( t \right)-d_{2}^{c}\cdot c_{2}\left( t \right)$ $\left( 6 \right)$

$s\left( t \right)=\frac{1}{1+k^{c}c_{2}\left( t \right)}$ $\left( 7 \right)$

$\frac{d}{dt}l_{1}\left( t \right)=2{\cdot a}^{l}{\cdot s\left( t \right)\cdot p}^{l}{\cdot l}_{1}\left( t \right)-p^{l}\cdot l_{1}\left( t \right)-d\left( x_{(t)} \right)\cdot l_{1}\left( t \right)-k_{chemo}\cdot p^{l}\cdot l_{1}\left( t \right)$ $\left( 8 \right)$

$\frac{d}{dt}l_{2}\left( t \right)=2{\cdot p}^{l}\cdot l_{1}\left( t \right)-{2\cdot a^{l}\cdot p}^{l}{\cdot s\left( t \right)\cdot l}_{1}\left( t \right)-d_{2}^{l}\cdot l_{2}\left( t \right)-d\left( x_{(t)} \right)\cdot l_{2}\left( t \right)$ $\left( 9 \right)$ x$\left( t \right)=$ $c_{1}\left( t \right)+$ $l_{1}\left( t \right)+$ $l_{2}\left( t \right)$ $\left( 10 \right)$

Introducing combination therapy into the model

In the following, we describe our rationale to refine and extend the model to also cover combination therapies. Intensive induction chemotherapy of AML contains a combination of two or more chemotherapeutics applied in a specific therapy regimen [5,6]. In our work, we focus on the widely used 7+3 schema based on 7 days cytarabine and 3 days anthracycline. Cytarabine acts as an antimetabolic agent and attacks primarily on cells during their synthesis phase (S-phase) by inhibiting the DNA-polymerase 𝛂 [7,8]. In this regard, the modelled chemotherapy can be considered as a cytarabine-like chemotherapy (now denoted by $k_{cyt}$) acting on proliferating cells.

In contrast to cytarabine, anthracycline affects not only proliferating but also non-proliferating cells via various mechanisms such as inhibition of topoisomerase II or free radical generation [9].

We expanded the model by a second type of chemotherapy acting additionally on non-proliferating cells (denoted by a constant factor $k_{anthra}$).
Hereby, by introducing both chemotherapies we now can model the effects of combination therapy on AML disease dynamics. For simplicity, we assume that the effect of anthracycline on mitotic cells is limited to the proliferation phase. We extended the initial model $\left( 5 \right)$ - $\left( 10 \right)$ to the following:

$\frac{d}{dt}c_{1}\left( t \right)=2{\cdot a}^{c}{\cdot p}^{c}\cdot{s\left( t \right)\cdot c}_{1}\left( t \right)-p^{c}\cdot c_{1}\left( t \right)-d\left( x_{\left( t \right)} \right)\cdot c_{1}\left( t \right)-k_{cyt}\cdot p^{c}\cdot c_{1}\left( t \right)-k_{anthra\cdot}p^{c}\cdot c_{1}\left( t \right)$ $\left( 11 \right)$

$\frac{d}{dt}c_{2}\left( t \right)=2\cdot p^{c}{\cdot c}_{1}\left( t \right)-{2{\cdot a}^{c}\cdot p}^{c}\cdot{s\left( t \right)\cdot c}_{1}\left( t \right)-d_{2}^{c}\cdot c_{2}\left( t \right)-k_{anthra}\cdot c_{2}\left( t \right)$ $\left( 12 \right)$

$\frac{d}{dt}l_{1}\left( t \right)=2{\cdot a}^{l}\cdot s\left( t \right){\cdot p}^{l}\cdot l_{1}\left( t \right)-p^{l}\cdot l_{1}\left( t \right)-d\left( x_{\left( t \right)} \right)\cdot l_{1}\left( t \right)-k_{cyt}\cdot p^{l}\cdot l_{1}\left( t \right)-k_{anthra}{\cdot p}^{l}\cdot l_{1}\left( t \right)$ $\left( 13 \right)$

$\frac{d}{dt}l_{2}\left( t \right)=2p^{l}{\cdot l}_{1}\left( t \right)-{2a^{l}\cdot s\left( t \right)\cdot p}^{l}\cdot l_{1}\left( t \right)-d_{2}^{l}\cdot l_{2}\left( t \right)-d\left( x_{\left( t \right)} \right)\cdot l_{2}\left( t \right)-k_{anthra}{\cdot l}_{2}\left( t \right)$ $\left( 14 \right)$

$x\left( t \right)=$ $c_{1}\left( t \right)+$ $l_{1}\left( t \right)+$ $l_{2}\left( t \right)$ $\left( 15 \right)$

$s\left( t \right)=\frac{1}{1+k^{c}c_{2}\left( t \right)}$ $\left( 16 \right)$

$d\left( x(t) \right)= {10}^{-10}\cdot max(0, x-4\cdot{10}^{9} cells/kg)$ $(17)$

The functions $s\left( t \right)$ and x$\left( t \right)$ were not modified and correspond to $\left( 7 \right)$ and $\left( 10 \right)$, respectively.

This model was implemented in the R statistical software package [10].

Table – Model parameters. Fixed values according to parametrization in [1]

| Parameter symbol | Description | Value |
| --- | --- | --- |
| $\boldsymbol{p}^{\boldsymbol{c}}$ | Proliferation rate of healthy hematopoietic cells (HSC) | 0.45 |
| $\boldsymbol{a}^{\boldsymbol{c}}$ | Self-renewal of HSC | 0.87 |
| $\boldsymbol{k}_{\boldsymbol{cyt}}$ | Cytarabine-like therapy intensity | Varied between 0-10 |
| $\boldsymbol{k}_{\boldsymbol{anthra}}$ | Anthracycline-like therapy intensity | Varied between 0-10 |
| $\boldsymbol{a}^{\boldsymbol{l}}$ | Self-renewal of leukemic stem cells (LSC) | Varied between 0-1 |
| $\boldsymbol{p}^{\boldsymbol{l}}$ | Proliferation rate of leukemic stem cells | Varied between 0-2 |
| $\boldsymbol{k}^{\boldsymbol{c}}$ | According to Stiehl 2014 | ($(2\cdot a^{c})-1$)/C_2_(0) |
| $\boldsymbol{d}_{\boldsymbol{2}}^{\boldsymbol{c}}$ | Apoptosis rate of HSC | 2.3 |
| $\boldsymbol{d}_{\boldsymbol{2}}^{\boldsymbol{l}}$ | Apoptosis rate of LSC | 0.1 |

Table – Initial condition for all simulations

| Compartment | Initial state |
| --- | --- |
| C1(t=0)* | $2\times{10}^{9}$ cells/kg |
| C2(t=0)* | $3.9\times{10}^{9}$ cells/kg |
| L1(t=0) | 1 cell/kg |
| L2(t=0) | 0 cells/kg |

* Compartment C1 and C2 start in the steady state of the healthy systems (without leukemic cells or therapy) .

To characterize the three selected leukemic clones we investigated the time from diagnosis to onset of complete remission (CR: bone marrow blasts < 5%).  Within the mathematical model the date of diagnosis corresponds to the beginning of induction chemotherapy as well. CR assessment is continuously performed starting from day 8 after therapy start independent from the applied induction regimen.

In addition to the time to onset of CR we examined the duration of occurred CR to characterize further properties of leukemic clones under the influence of chemotherapy.  In this process, we measured the time from the occurrence of CR to the last day of CR (either until the end of simulated days or until a leukemic relapse).

Chemotherapy schemata are implemented as events within the simulation program controlling the chemotherapy intensities in an on-off manner. During therapy the respective model parameters will be switched on to the predefined value or set to zero while no therapy should be applied.  Within the model therapy will start immediately at the selected chemotherapy intensities for a certain period, if blast fraction exceeds 20%.

**References**

1. Stiehl T, Baran N, Ho AD, Marciniak-Czochra A. 2014 Clonal selection and therapy resistance in acute leukaemias: mathematical modelling explains different proliferation patterns at diagnosis and relapse. J. R. Soc. Interface 11, 20140079–20140079.
2. Getto P, Marciniak-Czochra A, Nakata Y, Vivanco M dM. 2013 Global dynamics of two-compartment models for cell production systems with regulatory mechanisms. Math. Biosci. 245, 258–268.
3. Marciniak-Czochra A, Stiehl T, Ho AD, Jäger W, Wagner W. 2009 Modeling of Asymmetric Cell Division in Hematopoietic Stem Cells—Regulation of Self-Renewal Is Essential for Efficient Repopulation. Stem Cells Dev. 18, 377–386.
4. Stiehl T, Marciniak-Czochra A. 2012 Mathematical Modeling of Leukemogenesis and Cancer Stem Cell Dynamics. Math. Model. Nat. Phenom. 7, 166–202.
5. Döhner H, Estey E, Grimwade D, Amadori S, Appelbaum FR, Büchner T, Dombret H, Ebert BL, Fenaux P, Larson RA et al. 2017 Diagnosis and management of AML in adults: 2017 ELN recommendations from an international expert panel. Blood 129, 424–447.
6. Dombret H, Gardin C. 2016 An update of current treatments for adult acute myeloid leukemia. Blood 127, 53–61.
7. Cai J, Damaraju VL, Groulx N, Mowles D, Peng Y, Robins MJ, Cass CE, Gros P. 2008 Two Distinct Molecular Mechanisms Underlying Cytarabine Resistance in Human Leukemic Cells. Cancer Res. 68, 2349–2357.
8. Galmarini CM, Thomas X, Calvo F, Rousselot P, Rabilloud M, El Jaffari A, Cros E, Dumontet C. 2002 In vivo mechanisms of resistance to cytarabine in acute myeloid leukaemia. Br. J. Haematol. 117, 860–8
9. Sawyer DB, Peng X, Chen B, Pentassuglia L, Lim CC. 2010 Mechanisms of Anthracycline Cardiac Injury: Can We Identify Strategies for Cardioprotection? Prog. Cardiovasc. Dis. 53, 105–113.
10. R Core Team. 2014 R Core Team (2014). R: A language and environment for statistical computing. R Found. Stat. Comput. Vienna, Austria. URL http//www.R-project.org/. , R Foundation for Statistical Computing.
